# Instability and Uncertainty Are Critical for Psychotherapy: How the Therapeutic Alliance Opens Us Up

**DOI:** 10.3389/fpsyg.2021.784295

**Published:** 2022-01-07

**Authors:** Patrick Connolly

**Affiliations:** Counselling and Psychology Department, Hong Kong Shue Yan University, North Point, Hong Kong SAR, China

**Keywords:** psychotherapy process, therapeutic alliance, interpersonal synchrony, stochastic and deterministic stability, chaotic itinerancy, free energy principle and active inference (FEP-AI) framework

## Abstract

Tschacher and Haken have recently applied a systems-based approach to modeling psychotherapy process in terms of potentially beneficial tendencies toward deterministic as well as chaotic forms of change in the client’s behavioral, cognitive and affective experience during the course of therapy. A chaotic change process refers to a greater exploration of the states that a client can be in, and it may have a potential positive role to play in their development. A distinction is made between on the one hand, specific instances of instability which are due to techniques employed by the therapist, and on the other, a more general instability which is due to the therapeutic relationship, and a key, necessary result of a successful therapeutic alliance. Drawing on Friston’s systems-based model of free energy minimization and predictive coding, it is proposed here that the increase in the instability of a client’s functioning due to therapy can be conceptualized as a reduction in the precisions (certainty) with which the client’s prior beliefs about themselves and their world, are held. It is shown how a good therapeutic alliance (characterized by successful interpersonal synchrony of the sort described by Friston and Frith) results in the emergence of a new hierarchical level in the client’s generative model of themselves and their relationship with the world. The emergence of this new level of functioning permits the reduction of the precisions of the client’s priors, which allows the client to ‘open up’: to experience thoughts, emotions and experiences they did not have before. It is proposed that this process is a necessary precursor to change due to psychotherapy. A good consilience can be found between this approach to understanding the role of the therapeutic alliance, and the role of epistemic trust in psychotherapy as described by Fonagy and Allison. It is suggested that beneficial forms of instability in clients are an underappreciated influence on psychotherapy process, and thoughts about the implications, as well as situations in which instability may not be beneficial (or potentially harmful) for therapy, are considered.

## Introduction

Recent work by Tschacher and Haken (2019) reported in their book ‘The Process of Psychotherapy: Causation and Chance,’ offers an interesting perspective on a core healing mechanism of psychotherapy. Rooted within a perspective of systems science, the main idea communicated by their book is that change in psychotherapy can be described in terms of two processes, one *deterministic*, the other *chaotic*. A deterministic change process refers to a shift in a client’s behavior or experience in a particular (desirable) direction – for example, the client starts to experience generally more positive emotions. A chaotic change process refers to a greater exploration of the states the client can be in – in other words, during the course of therapy, the client may experience a wider range of thoughts, behaviors and intensities of feeling. In the model Tschacher and Haken (2019) provide, the ‘chaotic’ process is a necessary first step for therapeutic change. In the process they describe, the client first experiences a broadening of thoughts, emotions and behaviors, beyond their typical pattern or range (a chaotic process). Then the client’s states move toward a more desirable range of experiences (deterministic process), which in the final step, stabilizes into a new normal.

This idea of chaotic process is found in the science of complex systems, where it may also be referred to as a stochastic process (or sometimes, ‘disorder,’ ‘instability,’ ‘fluctuations,’ ‘itinerancy,’ or ‘noise,’ depending on context). In this field, the evolution and development of a wide variety of types of systems is determined both by deterministic tendencies toward order or stability, as well as tendencies toward disorder or instability. For systems that are living systems, stochastic processes can potentially be ‘beneficial’ (perhaps counterintuitively) for achieving their core aim, which, roughly, is to go on being living systems. This is because stochastic processes help facilitate adaptation (Ao, 2005). An example might be a new genetic mutation of a species, which tends to explore its environment more widely than earlier variants, may discover a new, much richly resourced environment, in the context of geographically distributed resources. The exploratory variant is more likely to broaden the geographical range around its resource center, or leave it altogether, which is formally analogous to escaping an ‘attractor’ state^1^. This role of chaotic processes causing a system to escape attractor states and find new ones is true of most systems that human beings study. Chaotic processes are also relevant to psychotherapy, not just in the sense that people can benefit from a wider range of behavior, but also in how our attention or conscious experience can similarly start to explore a wider range of possible perceptions of our environment, as well as a wider range of inner psychological experience, and escape being ‘stuck’ in a particular range of perception and experience.

While Tschacher and Haken (2019) offer some ideas about how psychotherapy facilitates beneficial stochastic processes in clients, this is not much developed in this work, which has more to say about the deterministic ones. In this article, the focus is on chaotic process, and a distinction will be offered here between a *general* state of increased (desirable) disorder *related to the quality of the therapeutic alliance or relationship^2^*, and a *specific* state of increased disorder triggered either by a particular *technique* used by the therapist, or by *circumstances* encountered by the client. Though some ideas are mentioned regarding *specific* states of disorder, the focus of this paper will be on developing a theory of how the therapeutic alliance results in a *general* state of instability, as a key element of a good therapeutic outcome.

To do this, the article will draw on Friston’s (2010) description of a hierarchical generative model as a way to understand the client’s self. While the workings of a hierarchical generative model will be explained later in this paper, the salient feature of the way such a model works is that control is exerted over how the system functions by ‘predictions’: messages that are passed down the hierarchy that also establish the ‘precision’^3^ (or certainty) applied to the environmental information passing upwards through the hierarchy. It will be proposed that the beneficial general state of disorder of the sort described earlier must rely on a broad relaxation (reducing) of the precisions passed downwards through the hierarchy, which has the effect of ‘opening the person up’ to information, which is now more able to change the hierarchical model. This will be explained in more detail later in the paper.

To understand how the therapeutic relationship may have this general effect on the precisions in the hierarchical generative model that is the client’s self, this article draws on theories about interpersonal synchrony and how it may entrain the client’s psychological, emotional and behavioral functioning (Koole and Tschacher, 2016; Tschacher and Haken, 2019). Friston and Frith (2015a) have developed a model that provides a consistent explanation of how interpersonal synchrony could result in such reduced downward precisions that are the focus of this paper. This will lead to a claim that a good therapeutic alliance or relationship (described in terms of effective interpersonal synchrony here) achieves a beneficial *general* disorder in the client, which is the starting point of meaningful therapeutic change.

It is suggested that Fonagy and Allison’s (2014) idea of mentalizing and epistemic trust in psychotherapy has a good consilience with the theory that is outlined here; specifically it will be highlighted that the operation of epistemic trust implies a destabilizing effect on the client’s self (though it may have a stabilizing effect later). This should offer clinicians some clear ideas about how the therapeutic relationship may facilitate a useful kind of disorder in clients. After pointing toward some limitations on the usefulness of instability in psychotherapy, it will be concluded that chaotic or stochastic processes are underappreciated in mainstream thinking in psychotherapy and in psychology more broadly.

## What Is Order and Disorder in Complex Systems?

The equations used by Tschacher and Haken (2019) to model psychotherapy are referred to as Fokker–Planck equations, and their purpose is to model both deterministic as well as stochastic processes in a system. This relationship of determinism and disorder in complex systems can be described by an analogy. If we were to imagine an object on a side of a valley we could describe the force of gravity as a deterministic force acting on it, always tending to push in one direction, which is down the slope of the valley toward the bottom. However, the actual movement of the object is also influenced by chaotic factors which could push it in either direction or let it come to rest^4^. Many types of systems can be described as a result of both of these factors, a deterministic pull in a particular direction (gravity in this example) while subject to random (or at least chaotic) ‘kicks’ in either direction.

**FIGURE 1:**
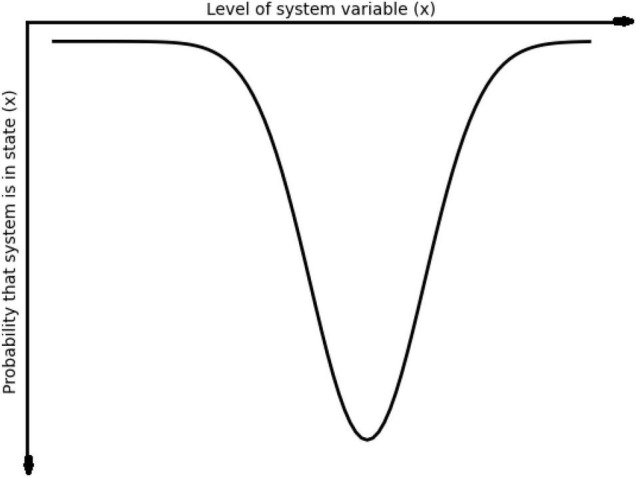
A basin of attraction (an inverted normal curve) following Tschacher and Haken (2019), indicating a distribution of probable states of the system variable (x) – or P(x), with the most likely state being at the bottom of the basin.

The valley in the analogy above could be described as a basin of attraction (represented in Figure 1). We could think of it as a normal distribution curve, turned upside down; the deepest point of the valley refers to the point that the system is most likely to inhabit, or spend the greatest amount of time. This basin of attraction then models the tendency of the system toward a particular stable state over time (the object tending to move toward the bottom of the valley), as well as indicating a range within which the system state tends to vary. The further the object from the bottom of the valley, the greater the likelihood that it will go down instead of further up; at the bottom of the valley, fluctuations can only ever push it upwards.

**FIGURE 2:**
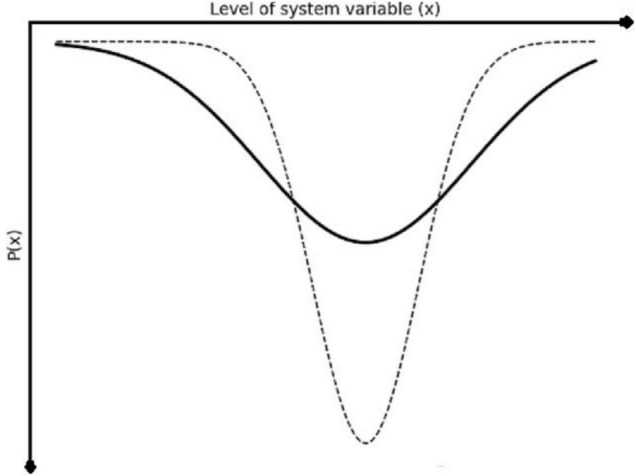
The solid line shows a shallow and wide basin of attraction, representing a system with more pronounced chaotic process (and reduced deterministic process) while the dotted line represents a system with less chaotic process and a more pronounced deterministic basin of attraction.

In this way of thinking about a system, the degree of chaos or disorder in the system may refer to how wide and shallow the basin is, as opposed to narrow and deep. Figure 2 shows a system that has a greater level of chaos, where the solid line refers to a system with higher disorder and lower stability (shallow and wide), and the dotted line refers to a system with lower disorder and higher stability (deep and narrow).

To use the analogy of the object in a real-world valley, a system of higher disorder means that there are a lot of different things going on in this valley that could push the object either up or down – or the valley is much shallower, meaning that the deterministic push of gravity toward the bottom of the valley has less influence relative to the more random fluctuations. In such a situation, we could imagine that the object could escape the valley, or even end up in another valley – which is really a description of one important way that systems change: through ‘*chaotic itinerancy*,’ or moving easily between multiple weakly attracting sets (very shallow basins of attraction, or valleys), and potentially ending in a new, stable attractor (Ao, 2005; Tyukin et al., 2009).

Chaotic itinerancy has been explored as a concept in a variety of phenomena related to neuroscience, cognitive science, robotics and artificial intelligence, where it has been connected to learning, adaptability and flexible information processing (Sadaghiani et al., 2010; Park et al., 2017; Northoff, 2018). An example is mind-wandering, which has shown to have potential benefits for creativity, problem-solving and future planning (Smallwood and Schooler, 2015; Zedelius and Schooler, 2015; Leszczynski et al., 2017; Metzinger, 2017).

## Applying the Fokker–Planck Model to Psychotherapeutic Change

Tschacher and Haken (2019) have applied the Fokker–Planck model to psychotherapy clients’ behavior in the following way. One could say that a client’s mental state, such as the level of positivity or negativity of their emotions, could be compared to the object on the slopes of the valley. This reflects a systems-based understanding of people’s emotions, which is useful in that it helps us understand how we may tend toward a particular pattern in our emotional life, such as how stably positive or negative our emotions tend to be over time. In this way, our emotions might vary in how positive or negative they are (the chaotic process), but they will tend toward a particular pattern or stable state (deterministic process).

**FIGURE 3:**
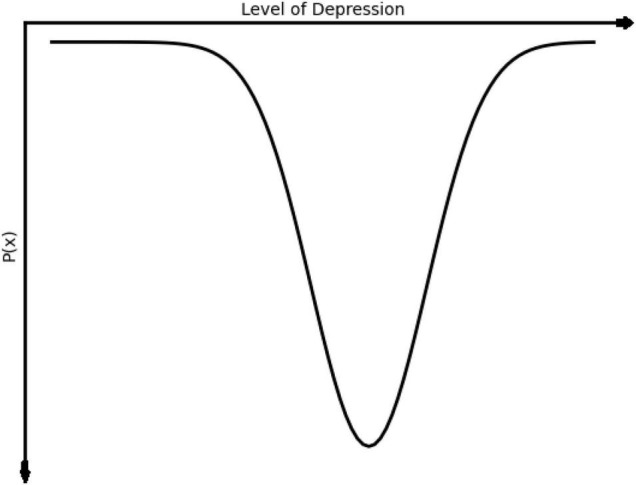
Idealized graph showing a typical attractor state of the level of negative emotion related to depression following Tschacher and Haken (2019). The goal of therapy would be to shift the mean of this function to the left toward more positive emotions.

This could be seen in the situation of a client with depression, which we can understand as a tendency to remain within a relatively narrow range of mostly negative emotions (represented in Figure 3).

The systems approach also offers a theoretical understanding of why and how people’s behavior does not change very easily, and the challenge facing psychotherapists (or psychiatrists) who would like to help facilitate such change.

However, through their application of the Fokker–Planck equation to states of the human system, Tschacher and Haken (2019) reveal an interesting theoretical implication for change in such systems that is very valuable for understanding psychotherapy process. This useful insight is to show how change in psychotherapy process can itself be understood as consisting of both determinist and chaotic processes. Deterministic change processes in therapy refer to factors (such as an intervention) that have the goal of shifting the basin of attraction in a more desired direction, or toward more stably positive emotion in the current example. Examples described by Tschacher and Haken (2019) in their book include cognitive interventions such as modifying cognitions to promote more positive emotions, or skills training in behavioral approaches, and others.

**FIGURE 4:**
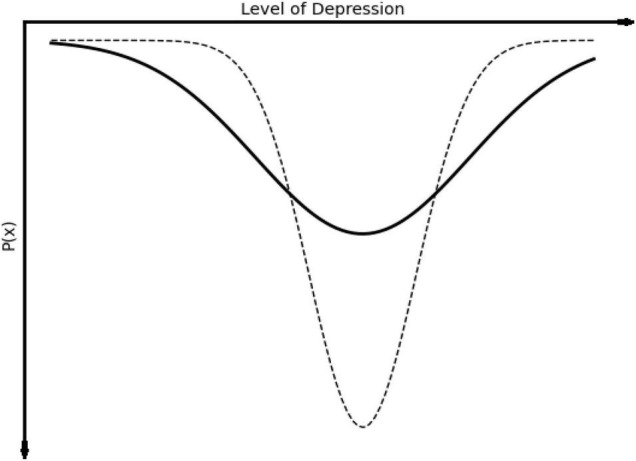
The stochastic change process (Tschacher and Haken, 2019), which involves a shift from a narrower, deeper basin of attraction to a wider distribution of system states, such as a wider range of emotional states, in either positive or negative directions.

For stochastic change processes in psychotherapy, Tschacher and Haken have suggested that techniques such as mindfulness, or free association in psychoanalysis, may be examples of interventions that do not push the system state in particular direction, but may have the effect of making a wider range of experience or behavior possible, as shown in Figure 4.

**FIGURE 5:**
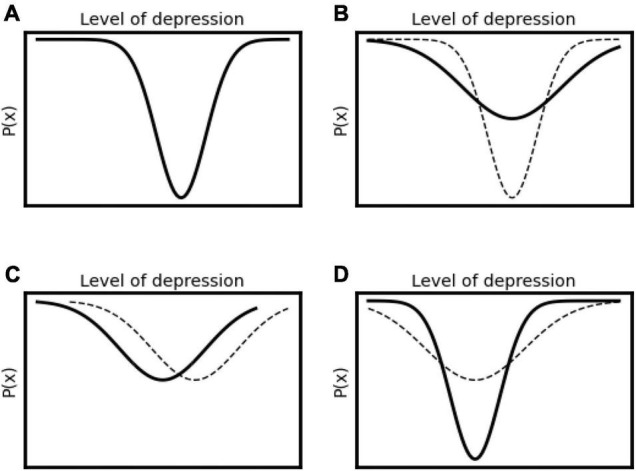
A model of stages of therapeutic change presented in Tschacher and Haken (2019). **(A)** Prior to change, a deep basin of attraction, reflecting here a relatively stable depressive state. **(B)** A first stage of change, being a stochastic (chaotic) process involving a wider range of system states. **(C)** A deterministic change in system states in a particular (desired) direction, here toward a more positive range of emotions. **(D)** A reduction in stochastic processes where the system settles into a new (more desired) stable state, here reflecting a stable range of more positive emotions.

Further, they have indicated that the change process in psychotherapy is likely to move forward in a particular order (indicated in Figure 5): first, the chaotic elements of change widen the basin of attraction of the system states, then deterministic elements of change move the basin toward a (hopefully) more desired direction, before it deepens again to become a new stable point of the system (through the self-organization processes of the system, or changes in contextual factors). In the example above, it may mean a depressed person starting to have a wider range of emotions, before starting to have a reduced level of depression, and this then becoming a new normal.

This theoretical work by Tschacher and Haken (2019) offers an important idea for therapists, which is that the first step of successful psychotherapy may be to facilitate beneficial ‘chaos’: a wider range of behavior (and experience) in the client, that is not necessarily only in the direction of desirable change, but genuinely *wider*, as a precondition for further change to take place.

This work also uses the same theoretical base as recent claims made for both brain stimulation, as well as microdosing psychedelics, for the treatment of depression. While the evidence for the effectiveness of microdosing psychedelics is not yet adequate (Kuypers et al., 2019; Polito and Stevenson, 2019), Carhart-Harris (2018) has proposed that the effectiveness of microdosing psychedelics as a route to reducing symptoms of depression, works through facilitating an increase in chaotic itinerancy in the nervous system (Carhart-Harris and Friston, 2019). Similarly, the effectiveness of brain stimulation on depression (Bermpohl et al., 2006) and other disorders has been explained through the concept of increases in spontaneous fluctuations of the brain as well (Northoff, 2018).

Where chaotic itinerancy is mediated through randomness, its positive effect on the functioning of the system has been formally described as simulated annealing, which is itself analogous to the process of annealing in metallurgy, where a metal is heated up over several occasions, becoming stronger in the process. By the heating process (either real or simulated such as by neural excitability^5^), the system reaches a state of greater plasticity, allowing it to settle again, this time in a new, potentially more optimal attractor state (Mehrabian and Lucas, 2006; Carhart-Harris and Friston, 2019).

While these proposed mechanisms for the efficacy of brain stimulation and microdosing psychedelics are broadly the same as that presented here, a difference might be in the hierarchical level of brain organization in which change is instigated; if brain stimulation or psychedelics succeed in increasing chaotic itinerancy, they likely do so in a bottom-up way in a hierarchical generative model, changing activities happening at lower, physiological levels (upregulation of 5-HT2AR serotonin functioning in the case of psychedelics) which then disrupt and shift control activities at a higher level (greater spontaneous cortical activity) (Carhart-Harris and Friston, 2019). In contrast, psychotherapy may have a more top–down influence, intervening as at a higher order of organization (instigated in the social interaction) which then results in increased fluctuations at a lower level of organization (including at physiological levels such as through body awareness in mindfulness). It is the purpose of the following sections to explain this concept of hierarchical brain organization, and how these various ways of instigating greater chaotic itinerancy in brain activity nonetheless have an equifinal endpoint which describes this fluctuation-inducing effect in the person, though there may be different roads to get there. This begins with a potential description of the stochastic process based on theoretical systems neuroscience, in the form of the free energy principle and the organization of hierarchical generative models in the brain, relevant aspects of which are introduced next.

## Precisions, Predictions, and Errors in Hierarchical Information Processing

Over the past decade-and-half, the free energy principle has become a very influential foundation for understanding brain and cognitive processes. It proposes that the organization of the brain entails a generative predictive model of the sensory inputs it receives, whether from outside the person, or somatosensory or interoceptive information from their bodies. In response to sensory inputs, the model actively generates inferences of the causes of those inputs, which inform a prediction of what information is expected next. The free energy principle then proposes that, as a system, the brain tends to minimize the difference between the sensory inputs that are predicted by the generative model based on its inference of causes, and what the received sensory inputs actually are; in other words it minimizes the prediction error. This tendency to minimize prediction error either takes the route of a Bayesian updating of the predictive generative model to be closer to the inputs it receives, or the system taking an action which brings the sensory inputs closer in line with what is expected (Friston et al., 2006; Friston, 2010). An example might be seeing an unexpected blemish on one’s skin: the error produced by this unexpected stimulus might be reduced by updating my expectation of how my skin appears, or rubbing the blemish with my hand in the expectation that the blemish might come off, thus preserving my prior expectation of how my skin appears (blemish-free).

**FIGURE 6:**
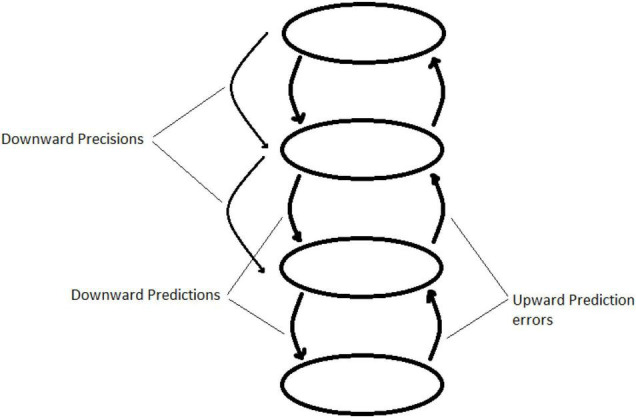
A hierarchical generative model which passes prediction error up the hierarchy while passing predictions (and precisions) downwards.

What is most important for the purpose of the current paper is the idea of a *hierarchy* in the generative model. The architecture of the brain can be described as a hierarchy of information processing (see Figure 6), where the sensory inputs enter at the lowest level (Friston, 2010). The information that is passed upward to the next level is the prediction error at that level, and so prediction error from each level passes information up the hierarchy. What is passed downwards, from the highest level down through each successive level down, are predictions: the generative model at each level essentially models the prediction error of the level below, and acts to minimize it, either through updating itself, or through an action which brings the input from the level below back to what is expected.

The value of this hierarchical arrangement is that it is capable of sustaining very complex (multi-level) generative models of the information that the brain takes in. The human brain contains numerically vast levels of hierarchy in its organization, with feedback processes within and between levels: this means that the above description is a highly simplified description of a complex reality, but that nonetheless allows one to make effective statements about broad tendencies within the brain’s workings.

The key importance that this hierarchical organization of the brain has for the current paper, lies in the way that the each of the levels of the hierarchy sets the ‘precisions’ of the predictions at the level beneath.

The statistical concept of ‘precision’ in this context refers to the level of confidence associated with a prediction. An example (similar to one found in Peterfreund and Schwartz, 1971) may that of the setting of a thermostat in an air conditioner. Such a thermostat works through maintaining a selected temperature. For example, if it is currently cooling a room, and a desired temperature of 26°C has been selected, it will allow a certain margin of variation around that selected temperature before taking action. If the margin is set to 1°C, then once the temperature goes above 27°, the air conditioner will turn on; should it drop below 25°, the air conditioner will turn off. A wider margin of 2°, will mean it turns on at 27° and off at 24, and so on. We could compare precision to this margin: high precision would be akin to a very small margin, reflecting a high confidence that the temperature will remain within a narrow range. This situation also leads to greater prediction errors with only minor variation in the state of the thermostat. In contrast, low precisions could be compared to a wider allowable margin of variation around the temperature, or a low confidence that the temperature will be 26° – larger variations won’t produce so much prediction error.

**FIGURE 7:**
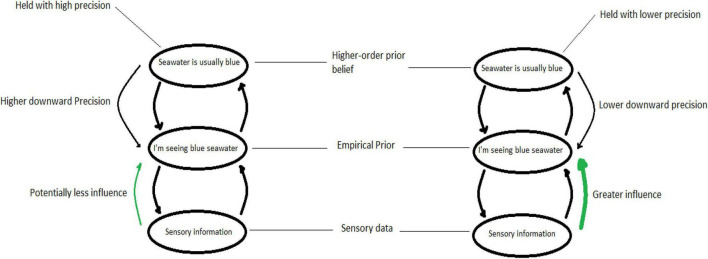
An idealized scheme showing a hierarchical generative model at three conceptual layers, a higher-order prior belief about visual scenes, and empirical prior applied to sensory information, and a sensory layer passing data upward. Where the higher-order prior is held with high precision, the empirical prior is also held with high precision, which can potentially take action to attenuate lower-level data from influencing higher levels. In contrast, where the higher-order belief is held with lower precision, the empirical prior is also held with lower precision, allowing low-level information to have a greater influence on higher levels (and thereby on overall system state).

Within a hierarchical free-energy principle framework, the precision associated with the predictions of a particular level of the hierarchy are determined in part by the precision with which prior beliefs at a higher level are held (Parr et al., 2018). To use an example (shown in Figure 7), if I have a very certain (high precision) higher-level prior belief that sea water always looks blue, then when I am looking at the sea right now, my prediction (empirical prior) that the color I am seeing is blue will be held with high precision too – and small variances from this prediction will generate error (and hence action or updating). Whereas, if my higher-level prior belief that sea water is blue is held with less certainty (lower precision), then my current prediction that the color of water I am looking at will be blue is also made with less precision, and generates less error – in this case the precision of the sensory input will be relatively higher, and what I will perceive is closer to the sensory information. This can still lead to updating, though to lesser degree, or to action though with lower probability.

The above section has described the role played in *perception* by the precision of higher-level prior beliefs; however, the selection and implementation of *action* can similarly be described in a hierarchy of precisions. Pezzulo et al. (2018) propose that the motivation aspect of action, which indicates our prior preferences for outcomes of our behavior, is also hierarchical, consisting of lower-level predictions of interoceptive signals (e.g., ‘I’m hungry’) and higher-level goals (e.g., ‘I should continue dieting’) founded upon episodic information, social incentives or higher-level prior beliefs about one’s self-image (these latter being quite important for the current paper). These motivations themselves set precisions for predictions in a control hierarchy which establishes policies of action that are associated with desired outcomes. For example, my prediction that the action I will take – that I won’t eat an unhealthy food – is likely to help me reach my desired state of being healthy (motivation) is likely to make me select that policy of action. These precisions, regarding the predictions that policies of action will produce desired outcomes, are themselves influenced by (the precision of) higher-level prior beliefs of the outcomes of policies.

This discussion of hierarchical processing (and particularly precisions) can now be connected back to the present discussion of psychotherapy process.

## Chaotic Change Processes as Reduced Precisions of Higher-Order Prior Beliefs

The discussion above suggests that the higher-order prior beliefs encoded within a client’s generative model, which give rise to predictions of its inputs (with active inference of its causes) as well as action selection and control and modeling the self, are held with particular levels of precision. This means, as a client, my higher-order prior beliefs that encode predictions about my environment, my body, my typical behavior, and particularly my personality, my values, my capabilities as a person, who I am and what I am doing, are held with a particular level of confidence.

The key suggestion of this paper is *that the chaotic process of change as described in Tschacher and Haken’s (2019) work may depend on a broad (or possibly even global) reduction of the precisions of higher-level prior beliefs in the hierarchy of the client’s generative model^6^*. A reduction in the precisions amongst these highest-level prior beliefs can be reached by various routes (top–down through a relationship, or bottom-up from within the person’s own nervous system), and such a reduction in precisions could result in meaningful changes in perception and behavior, and particularly, the possibility of a wider range of perceptions of behavior that might characterize the wider range of states associated with the chaotic (stochastic) process of change.

A conceptual example could illustrate how this would work. We could assume a high-level prior belief ‘I don’t really communicate much in groups’ held with high precision would influence (directly or through some intervening layers) the precision associated with my experience of my own behavior in group settings, and be connected with relatively consistent behavior of not communicating in groups. If the precision of this high-level prior belief were to become reduced, we would expect that the probability of selecting an action policy of not communicating in groups would also be reduced. This in turn would result in more inconsistency in the resulting behaviors: depending on other influences (interoceptive motivational influence, or contextual influences on control), the behavior selected would at times be not communicating, and at others choosing to express oneself. This widening in the range of behaviors (and in a similar way, perception) would appear like the shallower, wider basin of attraction in the chaotic process of change, and expresses the idea in Tschacher and Haken (2019) that chaotic processes are related to the permeability of boundary conditions of the system, and the extent to which it is affected by external factors.

In this way, we might expect that a client whose higher-level prior beliefs are held with reduced precision may subjectively perceive things they normally don’t, take actions they normally wouldn’t, have thoughts and emotions and express affect at times or in ways that are not typical for them, and experience an uncertainty about themselves and their identity that might hopefully feel good (or at least neutral) rather than distressing^7^. This would seem like an ‘opening up’ of the possible states of the client, and following Tschacher and Haken (2019), may precede a more deterministic shift of the system in a particular direction, and the formation of a new basin of attraction, in a more desirable state.

Certainly, this reduction in precision of higher-order beliefs in the generative model of the client may well be a necessary precursor to meaningful changes in it, and to allow for meaningful new learning to take place.

## How Reduced Precision of Prior Belief Results in Personal Change

Within a free energy principle framework, a generative model forms through recursive interaction with information from outside – its fair to say that it *is* a model of its environment, in a sense (Friston, 2010). This structural coupling of the generative model with its environment means that it is at the mercy of the environment to some extent. A generative model can only maintain a stable pattern to the extent that its environment allows for it^8^. In the case of a hierarchical generative model, we expect that the lower levels update more quickly, while higher levels may update more slowly – following the ‘slaving principle’ in Haken’s (1983/2004) ‘Synergetics.’ In fact, we might say that prior beliefs right at the top of such a hierarchy may remain relatively stable for significant phases of a person’s life; for example, a person’s personality (typical behaviors, for example) may seem relatively stable if their environment does not fluctuate too much.

The relative precisions in a hierarchical generative model may also play a role in maintaining the stability of higher-order prior beliefs. Parr et al. (2018) show how higher-order prior beliefs that are quite demonstrably false, can be maintained with a high degree of confidence (precision) through a reduction of the precision ascribed to sensory information (or information from lower levels of the hierarchy). In other words, we may ignore (or take other actions that ward off) information that will change our highest-level beliefs^9^ – in order to go on being the same person. Returning to an earlier example, sometimes we are committed (metaphorically speaking) to believing the sea is always blue, despite the evidence, and find any number of ways to avoid information that contradicts this.

This understanding of the stability of a hierarchical generative model also points toward how it can change. Essentially, the model is changed by new information. Sometimes that new information occurs when our environment changes – people often seek psychotherapy or counseling support when conditions in their life have changed, and it has become impossible to go forward being the person they are now – in this way crises bring change, often unwillingly.

However, psychotherapy attempts to facilitate change even when there is no major change in our environmental conditions. A reduction of the precision of high-level prior beliefs also creates the possibility for new information to be propagated within the hierarchy; in this way a person can have new experiences even in the absence of particularly novel information from one’s environment. Parr et al. (2018) show that if higher-order priors are held with low precision, then our current prediction (empirical prior) is more influenced by information from our senses – or from lower orders in the hierarchy. This new information does begin to update the generative model, although it may do so slowly (Parr et al., 2018). As described earlier, the relaxing of these high-order beliefs is likely to result in a wider range of perceptions and behaviors – which then provide a wealth of new information that can reshape and grow new beliefs at higher levels.

Friston (personal communication, 12 September 2020) suggests that basins of attraction of the sort shown in Figure 1 earlier in this paper, can also be considered free energy landscapes: deeper valleys refer to system states with high overall free energy, and wider shallower valleys depict systems with low overall free energy. A system that has high free energy is a system that is in an unlikely state (based on its prior expectations).

A fish out of water is such a system. In such a case there is a strong deterministic influence on its behavior: the most likely state dynamic^10^ the system is likely to have are actions that are predicted to bring it back into water, and other behaviors are not very likely. This state dynamic could be shown as a basin of attraction as well, where the state dynamic toward being back in water is the bottom of the basin. In contrast, a system with low overall free energy, is in a relatively likely state based on prior expectations. A fish in water, not particularly hungry, or without any meaningful demands on its behavior is an example. In this case, there may be no particular behavior that is very likely (besides respiration, which is expected), and so we may see greater chaotic itinerance (or exploration) in its behavior. This situation is analogous to a much shallower basin of attraction (as shown in Figure 2 above), where no particular system state (or state dynamic) is much more like than others.

Systems with low overall free energy (shallower basins) are in a state that lends itself to development and adaptation (a state referred to as self-organized criticality, Friston et al., 2012). He states:

*“In this shallow valley, one can*… *meander and explore with impunity – leading to long-term correlations of the sort seen in self-organized criticality (c.f., the difference between a mountain stream following a V-shaped valley and the meandering course of an estuary over a flat landscape).” (Friston, personal communication*, 12 September 2020*).*

He suggests that the tendency of biological systems to reduce free energy effectively means that there is an innate drive to find shallower landscapes, and therefore develop and adapt (self-organize).

A more human example might be a state of extreme psychological dependence in a relationship. If a person expects the near-constant presence and reassurance of a romantic partner, this is likely to be a high free energy system, that produces a strong deterministic influence on their thoughts, affect and behavior, which will all likely be directed toward securing and maintaining the presence of the other person, while other exploratory behaviors are much less. In contrast a person who is much less dependent on their relationship would be in a lower free energy state, and have a much weaker deterministic influence on their behavior, which would make exploratory behaviors (unrelated to the relationship) much more likely. They may be more likely to have varied activities, develop hobbies and friendships, or possibly leave the relationship.

A range of psychiatric conditions have also been viewed as a problem of such adaptation, cast in computational psychiatry literature as the result of overly precise prior beliefs (Van de Cruys et al., 2014) which result in high free energy states, with strongly attracting state dynamics. Abberant prior precisions, and beliefs about prior precisions have been proposed as key to explaining features of depression and mood (Seth and Friston, 2016; Badcock et al., 2017; Clark et al., 2018), schizophrenia and delusion (Tschacher et al., 2017) and aspects of obsessive compulsive disorder (Kiverstein et al., 2019), amongst others.

The question of how psychotherapy achieves a reduction in precision of higher-level priors, and allow adaptive development in the person, is addressed next.

## How Psychotherapy Reduces the Precision of High-Level Prior Beliefs

Psychotherapy is not the only route to meaningful personal change. Significant changes in our environmental circumstances (or our anatomy) have the potential to radically alter the information that shapes us, though the full extent of change may take some time to unfold. Tschacher and Haken (2019) describe these as changes in contextual influences or affordances. A change in circumstances, such as a crisis or an opportunity for a meaningful reward (e.g., employment and a living wage to someone who has been struggling), can have significant transformative capacity on self-understanding and behavior.

In the psychotherapy situation, various techniques can deliberately facilitate stochastic influences on change. These techniques were referred to as facilitating *specific* states of disorder earlier in this paper. Tschacher and Haken (2019) have mentioned free association in psychoanalysis, or mindfulness training, as techniques of this kind. Holmes and Nolte (2019) have specifically explored this influence of free association in terms of a hierarchical generative model in a free energy principle framework:

*“In free association, thoughts, interoceptive bodily sensations and affects, impulses and images enter the mind ‘from below’. As analysand and therapist collaboratively enter states of free-floating attention and negative capability, top-down constructions are temporarily set aside.” (p.19)*

In this way, free association seems to rather directly request the client to reduce the influence of higher levels of the hierarchical generative model (while the therapist does likewise), allowing more information to emerge from below, allowing new experiences and change.

Likewise, mindfulness techniques emphasize an acceptance of stimuli and mental states while reducing action taken to alter that information, similarly allowing an increase in the permeability of boundary conditions at the highest levels of the hierarchy. As Tschacher and Haken (2019) point out, there may be quite a number of techniques that explicitly produce chaotic change processes, while many techniques in psychotherapy may influence both deterministic and chaotic change processes at the same time.

The central focus of the remainder of this paper is not on these routes toward therapeutic uncertainty, but is rather on the role of the relationship itself as the most fundamental driver of chaotic or stochastic process, which I have referred to as a general state of disorder to distinguish its influence from that created by specific techniques. While Tschacher and Haken (2019) imply that the relationship may have influences on both stochastic and determinist change, their book discusses the role of the psychotherapy relationship more in terms of indirect or contextual determinist influences. The use of a hierarchical generative model approach within a free energy principle framework, has offered a conceptual bridge to make sense of the contribution of the relationship to stochastic change, and how it may indeed be a first and necessary step toward change. A description of the way in which the relationship can facilitate this general state of disorder is next.

### Relational Synchrony, Entrainment, and High-Order Priors

It is a long-standing idea in systems theory in psychology (Bateson, 1972; Keeney, 1983) that social interaction generates a recursively new step in the organization of the human psyche and behavior: in short, a relationship between two people (a system consisting of two interacting components) comes to pattern the emergence of the next higher layer of organization in the person’s mind and behavior. Haken’s (1983/2004) development of the study of Synergetics placed these ideas of the emergence of self-organization in systems on a clear formal footing, and his work fundamentally informs Tschacher and Haken’s (2019) approach in their book. A central idea here is the slaving principle, which is a form of self-organization in systems where slower ‘macro-processes’ in a system tend to entrain faster ‘micro-processes.’ While this was originally applied to problems in physics, it has subsequently been applied to problems in a wide variety of sciences, including several in psychology, and most relevantly here, psychotherapy (Schiepek et al., 2016). Key to the process of self-organization is the role of synchrony within systems – as fast-microprocesses synchronize with each other, a new, slower, macroprocess emerges which henceforth entrains those microprocesses (Haken, 1983/2004).

The importance of the emergence of interpersonal synchrony has informed a growth of research in psychotherapy process (as well as interpersonal interactions in general) that observes that synchrony does spontaneously emerge in interactions, that it is associated with more positive reports of the quality of interactions and the outcome of therapy and that it may operate on diverse levels, such as physiological (e.g., heart rate variability and skin conductance), cognitive, and affective, and that there may be a progression from more basic physiological levels toward cognitive and then emotion-complex levels of synchrony in successful therapy (Koole and Tschacher, 2016).

Tschacher and Haken (2019) model psychotherapy process in a way that suggests that it is the stability of the therapist’s states (such as their degree of mindfulness and calmness) that entrains the client’s states through synchronization, providing a deterministic shift in the client states (such as anxiety) which therapy seeks to change.

However, one perspective from a free energy principle framework offers a slightly more nuanced understanding, which suggests that it is not so much the therapist’s states that directly entrain the client’s states, but rather the relational system that forms between the two that entrains the client’s (and indeed therapist’s) states, though the therapist’s states clearly influence that relational system. Friston and Frith (2015a; 2015b) show how generalized synchrony in the form of an attractor manifold emerges when two agents (in their example, synthetic birds learning to sing in tandem) attempt to minimize their own free energy through bringing their own model of the birdsong closer to the model of the other, until they are ‘singing from the same hymn sheet,’ as the authors suggested. The emergence of this synchrony involves a dissolution of boundaries between the two participants where the sensory input from one becomes the prior of the other, as if it emerged from within.

The development of the therapy relationship can be approached in the same way, as the development of a synchrony (and dissolution of boundaries) between therapist and client, which comes to organize the experience and behavior of both of them. This situation supports ideas from intersubjective theories of the therapy relationship (Stolorow et al., 1994), which view the relationship as its own entity which is nonetheless constructed by both client and therapist. In other words, successful synchrony in therapy must entrain both client and therapist, though the therapist should hopefully have a meaningfully strong positive influence on the relationship.

Friston (personal communication, 12 September 2020) then clarifies how this emergence of interpersonal synchrony entails chaotic or stochastic processes in a general hierarchical model:

*“The role of precision is perhaps most clearly evinced – in the setting of dyadic synchronization – in the simulations of Friston and Frith (2015b). In these simulations of active inference (under the free energy principle) two birds sing and listen to each other under distinct internal or generative models of their shared song or narrative. It had been previously shown that when two members of a dyad share a generative model, generalized synchrony or synchronization of chaos is a necessary and emergent phenomena (Friston and Frith, 2015a). However, when the dyadic pair have different generative models, they can learn from each other and converge upon a (generalized) synchronization manifold. Crucially, the rate at which each member approaches this manifold depends upon the precision or confidence placed in their high-level (prior) beliefs. The numerical analysis in Friston and Frith (2015b) shows that the bird with the higher precision – or conviction in her model of the world – effectively teaches the other bird, such that their generative models converge. At this point, there is mutual inference and predictability, such that they are both ‘singing from the same hymn sheet.’ This was a proposal for resolving the hermeneutic problem; namely inferring the intentional stance of another. Mathematically, it is simply a manifestation of generalized synchrony, under the free energy principle.”*

While Friston and Frith’s (2015b) work here focuses on how interpersonal synchrony entrains fast scale interpersonal processes (that pattern perceptual inference), the important conclusion here is that through the development of the person over time, the person’s generative model is organized by significant relationships over longer timescales, through new structural learning. Support for this perspective has come from work by Feldman (2012) on biobehavioral synchrony in parent-infant interaction, which has demonstrated that interpersonal synchrony observed in parent–child interactions has been linked with better attachment and parent–child bonds, as well as general socio-emotional development, as well as self- and co-regulation of affect.

This hierarchical nature of our generative models is critical to the current discussion, because it allows us to consider the apparent paradox where the seeming stability offered by the relationship nonetheless *increases* stochastic processes in the client to facilitate chaotic processes of change. This is because, during the development of a person, new layers are added to a hierarchical generative model through recursive self-organization in the hierarchical generative model, which are in turn organized by the relationships. Essentially, as the new highest level of the generative model emerges and gains stability (patterned by a relationship, for example), so the overall complexity of the self (and other) that can be modeled by the system increases as well. Since this increased capacity of the client to model their states lowers the overall free energy of the client as a system, so the imperative to maintain a particular system state should be reduced as well, and with it, the precisions associated with the priors at lower levels in the hierarchy as well. In this way, stability at the highest level can create or facilitate instability at lower ones^11^. This follows a principle in organization of a hierarchical generative model in that the precisions at one level are determined by the level of complexity at the level above. Returning to the example from earlier in the paper, if the higher-order prior beliefs regarding the color of sea-water in visual scenes become more complex (i.e., that water can be darker or lighter blue, or green, or gray or various shades) it means that the downward precision associated with the empirical prior below is lower, allowing a greater influence from sensory information below. If the higher-level prior was simpler (i.e., sea water is always blue), the downward precision would tend to be higher.

This is relevant to the present discussion as follows. Before any change process is initiated by the person before they come to therapy (or experience major changes in their environment or body), we would expect that the highest-level priors (for example, ‘who I am’) reflect the limits of the organization present in the person’s generative model. The addition of a new higher layer (in this case the formation of the stable relationship which entrains the client’s states and starts organizing the emergence of further higher levels) should have an effect similar to making that new higher level more complex – provided it allows for some complexity in modeling the client’s generative model. It should allow those previously highest-level priors to be held with less precision, and therefore to allow lower-level information greater influence over system states – which is the chaotic or stochastic process described above. In this way, the relationship^12^ may open the client to information, leading to a widening range of behavior and experience we might expect from the chaotic or stochastic elements of change.

### Consilience With Fonagy and Allison’s Concepts of Mentalizing and Epistemic Trust

There is a good consilience in the process described in this paper, with the work of Fonagy and Allison (2014) on the mentalization function in the therapy, and epistemic trust in the relationship. In their work, mentalizing refers to the activity where the therapist experiences the mental states of the client and reflects this experience to the client (or a caregiver to a child), providing the client an organizing experience of their inner life which is critical to development and therapeutic progress. Epistemic trust (of a client, for example) can be described as the relaxation of epistemic vigilance and an openness to learning from another person, that occurs in the context of a relationship whether, for example, a child in an attachment relationship with a caregiver, or a client in a relationship with a therapist. Fonagy and Allison suggest that epistemic trust is more likely to develop toward another person, when both the verbal and non-verbal communication from that person is experienced as relevant to oneself.

There may be many aspects of a client’s experience of a therapist that makes their communications seem relevant to us, such as racial and cultural similarity (Ward, 2005), attractiveness and personal similarity (Nerison and Claiborn, 1990) and other characteristics. However, regarding the trainable conduct of the therapist, Fonagy and Allison’s paper implies that a therapist’s communication is most likely to be relevant to a client when the therapist is accurately mentalizing the inner states of the client, which is a precondition for higher-level synchronization in therapy, or two minds doing the same thing at the same time, as described in Friston and Frith (2015b) or similar to what Mergenthaler (2008) called the Resonating Minds theory of psychotherapy, or Koole and Tschacher’s (2016) integrative framework for the therapeutic alliance.

Fonagy and Allison (2014) suggest that the trust that forms relaxes the ‘epistemic vigilance’ of the client, in doing so making them more open to learning and to a wider range of experience. This may counteract a process of epistemic freezing (described by Kruglanski, 1989) where a person maintains or defends their existing knowledge structures, even when they create adaptive problems for the person.

Closer to the systems perspective taken in this paper, Schoeller et al. (2021) have formalized trust within a predictive coding framework. In their study they have formally described trust as an agent’s best explanation for reliable sensory exchange with a partner, or where our prediction of the outcomes of our behaviors toward a partner are held with high certainty. In plain words, we might say that trust in this context is a situation where people respond to us the way we hope they will. In the case of therapy, this may often refer to the client’s hope that a therapist will listen to and understand them, not judge them and feel positively about them, or possibly guide them in some sense. While the definition of trust developed by Schoeller et al. (2021) veers closer to control than that in psychotherapy literature (their study is focused on human-robot collaboration), the key foundation is the same, that the client trusts a person whose behavior seems to reliably support the clients goals or aims (or is relevant to them, in Fonagy and Allison’s sense).

Within the approach taken by the current paper, the client’s ‘opening up’ to knowing, experiencing and learning as a result of trust in the relationship (in the form of synchronized states described in Friston and Frith, 2015a), and its capacity to organize the emergence of a new level of organization of the person’s generative model, and the resulting broadening of the client’s system states, is formally similar to and compatible with the systems-based description being offered in this paper.

### Chaotic Process as a Necessary Condition of Therapeutic Change

Tschacher and Haken (2019) have modeled chaotic processes as a necessary first step in psychotherapeutic change^13^. The implication here is that a reduction in precisions of high-level prior beliefs may be a necessary condition of meaningful change in psychotherapeutic relationships. While Parr et al. (2018) show that higher-order sensory priors held with high precision can update quickly when conflicting with sensory information, this cannot be true of the prior beliefs at the highest levels of the hierarchy, since by definition, these are the ones that change slowest – they are most likely those that predict or model the self (Palacios et al., 2020), which are often the target of therapeutic focus^14^. They have formed and are maintained because the relative plasticity or affordance in the environment permits their maintenance, and in the absence of major environmental change or significant development, are difficult to alter, due to the generative model’s tendency to maintain its own organization as far as possible (Connolly, 2016; Hohwy, 2016), even to the extent of repressing meaningful levels of information to do so (Hopkins, 2016; Connolly, 2018). In this context, unless there is significant change in the person’s environment (or anatomical processes, or meaningful structure learning) which makes it impossible to continue maintaining these beliefs^15^ (as mentioned above this is often the source of crisis which make people seek help in psychotherapy), a weakening of the precisions associated with them, and the subsequent increase in available information is the only way that meaningful (permanent) therapeutic change may take place^16^. In the absence of major contextual (or anatomical) change, a reduction of precisions of highest-order beliefs is a *necessary* condition of meaningful personal change.

This necessary condition may underlie the most fundamental finding from process-outcome research into psychotherapy over most of the last century, which is the centrality of the therapeutic relationship in facilitating change in psychotherapy. This has been largely conceptualized as the therapeutic alliance (or related terms) in literature, and has been supported by hundreds of studies in psychotherapy as well as a number of major reviews of this literature (Llewelyn et al., 2016). Given the perspective being outlined in the current paper, should the relationship fail to establish the necessary synchrony (that can entrain the client’s states), the instability at lower levels (through reduced precision associated with higher-order priors) never emerges either, leading to limited or failed outcomes in psychotherapy. Some support for this comes from the Vanderbilt studies of behavior in psychotherapy conducted by Strupp (1993) which identified the problem where clients who had negative or hostile views toward the therapist from the start, simply never had a positive outcome from therapy, and often ended up triggering negative or hostile responses from therapists themselves, typically by the third session^17^. Similar to Fonagy and Allison (2014) we might say that if a client does not trust the therapist enough (or more formally, achieve meaningful interpersonal synchrony with them) to step away from the certainty of their self-beliefs, or their conviction about how to approach life, the ‘opening up’ of stochastic change processes may never happen.

### Specific Instabilities in Psychotherapy

So far, this paper has focused on a very fundamental (general) form of instability in the service of change, one that is linked with the forming of a special form of relationship. It is one that can last through weeks or even months of psychotherapy, or longer. While it may be decisive in terms of whether psychotherapy can progress or not, it is likely also on a continuum of intensity (just as the relationship can have a degree of stability or synchronicity).

However, there are certainly fluctuations in the level of intensity of instability during the course of therapy, just as there are (perhaps related) shifts in synchronicity or cohesion. The idea of instability as a precursor to change is already well-established in psychotherapy, though often it is viewed in terms of faster, intra-session processes (or even microprocesses), that are much more short-lived, and possibly more intense. It is based on an established idea in systems studies, that systems may go through periods of critical instability ahead of phase change before reaching a new stable state (Haken, 1993; Schiepek et al., 2016). This critical instability has also been proposed as a crucial element in psychotherapy process, that has acquired empirical support in analysis of time series from behavioral, physiological, and neurological data (see Schiepek et al., 2016, for an overview), as well as in linguistic analysis of psychotherapy transcripts (Mergenthaler, 2008; Walter et al., 2010).

While such critical change periods in psychotherapy may arise spontaneously in interaction, they are not inevitable even within the context of a good therapeutic alliance, and may often be related to processes within sessions. Research on the precursors or causes of such states has not yet given any clear answer (Schiepek et al., 2016). Certainly, several techniques in psychotherapy may intentionally try to create these states, such as confrontations – whether they are in the service of setting up interpretations (Greenson, 1967), or challenging core beliefs or evidence (Beck et al., 1979) – or gestalt techniques that escalate current sensory experience at the expense of established patterns of behavior (Perls, 1969), or the initial stages of the focusing (Gendlin, 1996), or many others. Despite their difference from the longer-term instability described in this paper, in terms of the current formulation their therapeutic value may well be the same in the sense of acutely weakening prior beliefs in order to allow a new and transformative experience, thought or behavior at that moment in the session.

### When Instability Is Desirable or Undesirable in Therapy

Up until this point, the focus has been on the positive role that chaotic or stochastic change processes can play in psychotherapy. Clearly, such therapeutically induced uncertainty or instability is helpful in situations where stability is the problem, in terms of perceptions, behaviors or beliefs that are difficult or slow to change (e.g., certain forms of depression). However, there are clearly situations where instability is either undesirable or potentially harmful, in which therapeutic activities that activate chaotic processes should be avoided or at least mitigated or compensated for.

A clear example is in the case of psychotic disorders or a general weakness in reality testing. Tschacher and Haken (2019) highlight this treatment concern in their description that psychiatric facilities that treat patients with schizophrenia and psychosis actively try to maintain stability and reduce chaotic process by limiting disturbance and information from the environment. For this reason, very challenging techniques, or those that deliberately try to disrupt thought processes such as gestalt techniques may be harmful in work with clients with chronic psychotic symptoms^18^.

Another limitation applies to cases where the person’s inner states are already unstable, and their current need is toward reestablishing stability. Clients in the midst of crisis, or facing overwhelming environmental pressures, or having recently experienced trauma, are already experiencing chaotic change processes instigated by profound disruptions in the environmental context and their experiences, and the highest levels of organization of their self are already destabilized and under threat. As stated earlier in the paper, these disruptive situations have the potential for change in a positive direction (the phenomenon of post-traumatic growth), though this is not a certainty. Regardless, such clients are already in a condition of high instability, and are usually more in need of support than even greater instability. Some studies have in fact found potential harmful outcomes of therapy in the period shortly after a traumatic event such as Critical Incident Stress Debriefing (Carlier et al., 2000; McNally et al., 2003).

Finally, an ethical consideration applies as well. The theory developed in this paper offers an explanation for the vulnerability of clients who have opened up to the therapeutic relationship, and that it may work through the relative loss of precisions of their own beliefs. It also explains how clients can be taken advantage of by unethical therapists who hope to keep clients in therapy by facilitating the client’s uncertainty in their own beliefs.

While Tschacher and Haken’s (2019) insights regarding chaotic change processes could be applied to understanding psychotherapy in various ways, one that seems immediately relevant is in terms of assessing a client’s need in terms of stability or instability, which may differ not only from client to client, but also from session to session and moment to moment in therapy.

### Implications for Therapeutic Practice and Research

The key implication for psychotherapists from Tschacher and Haken’s (2019) work that is relevant to this paper, is that instability and uncertainty may be critical and helpful for psychotherapeutic change, whether it emerges spontaneously such as through the relationship, or through directive techniques of the therapist to induce instability. It may be important for therapists to recognize stochastic process when they see it, with an understanding of its potential benefits and risks.

Regarding techniques, while it may be that new techniques may be described in future work that specifically induce stochastic processes, it is perhaps more immediately relevant for practitioners to recognize the stochastic effects of techniques that are already well established in psychotherapy literature (a number have already been described in this paper and in Tschacher and Haken’s) and to be more intentional in using them with this effect in mind. This includes learning to how to manage instability when it appears, how to capitalize on it for the best effect, and selecting when to facilitate it and let it run, and when (and how) to try restore stability if one can.

While processes of instability are already well-observed in psychotherapy (Schiepek et al., 2016), Tschacher and Haken (2019) suggest that advances in understanding of chaotic processes is needed and is at an early stage. Certainly, the present paper points toward whether the establishment of the relationship itself may be linked to greater beneficial instability in client states (independent of deliberate techniques that facilitate instability), and considerations for evaluating instability responses following deliberate techniques that attempt to facilitate it. Work on stochastic process may also play a role in examining harmful outcomes in psychotherapy, where a client’s priors may be scrambled by destabilizing therapy practice, with no move toward rebuilding stability in the client.

## Author Contributions

The author confirms being the sole contributor of this work and has approved it for publication.

## Conflict of Interest

The author declares that the research was conducted in the absence of any commercial or financial relationships that could be construed as a potential conflict of interest.

## Publisher’s Note

All claims expressed in this article are solely those of the authors and do not necessarily represent those of their affiliated organizations, or those of the publisher, the editors and the reviewers. Any product that may be evaluated in this article, or claim that may be made by its manufacturer, is not guaranteed or endorsed by the publisher.

## Footnotes

1 In this case, the attractor state is the behavior of maintaining distance from a resource center, within a a bounded geographical range.

2 Following Bordin (1979) ‘therapeutic alliance.’

3 A detailed definition of generative models, predictions and precisions is offered later in this paper, in the section ‘Precisions, predictions and errors in hierarchical information processing.’

4 We should note that chaotic processes are not necessarily actually random – various deterministic influences can act on the object (we could also say there is structure in the noise). Besides being mediated by randomness, chaotic processes may be also be mediated by deterministic (but conservative) flows in a system.

5 Or by the metaphoric ‘warming’ due to the relationship, as described later in this paper.

6 The question of which priors are so altered is addressed later in this paper.

7 The reason it may not be experienced as distressing is discussed a little later in this paper.

8 This is related to the concept of affordance, that our environment allows particular patterns of behavior.

9 Hopkins (2016), has proposed a mechanism like this to explain the emergence and maintenance of an unconscious in a person. It may similarly describe maintenance of unsymbolized incongruent experience in line with Rogers (1959) theory of personality, or Beck et al. (1979) cognitive errors that maintain distorted schemas.

10 The trajectory of the system over time.

11 A common idea in levels analysis that has been offered as an explanation for a variety of phenomena, such as the stability–instability paradox (Rauchhaus, 2009) that suggests that the high-level peace between nuclear capable countries is related to an increase in lower-level conflict and instability. As an example of how this could work in the current discussion, consider how the securely attached child nonetheless exhibits a wider range of behaviors and exploration than the insecurely attached one in the strange situation experiments (Ainsworth and Bell, 1970) – the stability of the relationship is related to greater variance in behavior.

12 Besides the relationship, the process of *self-reflection*, whether in the context of a relationship or not, may be conceptualized as similarly adding a new layer to the generative model, with precisely the same stochastic effect that is described here, though the stochastic influence of the relationship should make it more effective, and supported by the mentalizing action of the therapist – see Fonagy and Allison (2014) -which is discussed later in this paper – for a description of mentalizing in therapy.

13 My thanks to Dr. Fabio Tha, who has suggested that the chaotic process is likely to be recursive, and that the new organization continues to update throughout the therapy process.

14 This holds true at higher orders of organization as well. Lakatos (1970) proposed philosophy of the history of science, which suggested that the most peripheral beliefs of a theory are relatively easy to change, but the core of the theory is not, and it changes much more slowly. Certainly, from the perspective of hierarchical recursive organization, social and cultural practice of science represent even slower, higher-order processes, presumably even less changeable than individual generative models.

15 An example may be a traumatic event that ‘shatters the assumptions’ of safety and predictability of the world (Janoff-Bulman, 1989) or the loss of a very significant other, or sudden poverty and loss of income (though the resulting change in the higher-order beliefs in the form of adaptation or grief also takes a long, painful time to unfold in these cases as well).

16 Mathys et al. (2011) demonstrate that learning is related to the level of precision-weighted uncertainty in a hierarchical generative model.

17 This is not to contradict the idea that overcoming hostility is very valuable for the client (such as interpreting negative transference), but rather that where the client has never had any positive feelings (transference) toward the therapist, therapeutic progress may be extremely difficult, or take a different form to the key processes described in this paper.

18 A helpful distinction may be Kernberg (2004) description of neurotic vs. psychotic personality organization: disorders related to neurotic organization (such as depression) characterized by excessive ego defense (for which instability may be therapeutically helpful) and disorders of psychotic organization reflecting poor ego strength and development (for which instability is more likely to be destructive).

## References

[B1] Ainsworth M. D. S. Bell S. M. (1970). Attachment, exploration, and separation: illustrated by the behavior of one-year-olds in a strange situation. *Child Dev.* 41, 49–67. 10.2307/1127388 5490680

[B2] Ao P. (2005). Laws in Darwinian evolutionary theory. *Phys. Life Rev.* 2 117–156. 10.1016/j.plrev.2005.03.002

[B3] Badcock P. B. Davey C. G. Whittle S. Allen N. B. Friston K. J. (2017). The depressed brain: an evolutionary systems theory. *Trends Cogn. Sci.* 21, 182–194. 10.1016/j.tics.2017.01.005 28161288

[B4] Bateson G. (1972). *Steps to an Ecology of Mind: Collected Essays in Anthropology, Psychiatry, Evolution, and Epistemology.* Chicago: University of Chicago Press.

[B5] Beck A. T. Rush A. J. Shaw B. F. Emery G. (1979). *Cognitive Therapy of Depression.* New York, NY: Guilford Press.

[B6] Bermpohl F. Fregni F. Boggio P. S. Thut G. Northoff G. Otachi P. T. (2006). Effect of low-frequency transcranial magnetic stimulation on an affective go/no-go task in patients with major depression: role of stimulation site and depression severity. *Psychiatry Res.* 141 1–13. 10.1016/j.psychres.2005.07.018 16352348

[B7] Bordin E. S. (1979). The generalizability of the psychoanalytic concept of the working alliance. *Psychother. Theory Res. Pract.* 16, 252-260. 10.1037/h0085885

[B8] Carhart-Harris R. L. (2018). The entropic brain – revisited. *Neuropharmacology* 142 167–178. 10.1016/j.neuropharm.2018.03.010 29548884

[B9] Carhart-Harris R. L. Friston K. J. (2019). REBUS and the anarchic brain: toward a unified model of the brain action of psychedelics. *Pharmacol. Rev.* 71 316–344. 10.1124/pr.118.017160 31221820 PMC6588209

[B10] Carlier I. V. E. Voerman A. E. Gersons B. P. R. (2000). The influence of occupational debriefing on post-traumatic stress symptomatology in traumatized police officers. *Br. J. Med. Psychol.* 73 87–98. 10.1348/000711200160327 10759053

[B11] Clark J. E. Watson S. Friston K. J. (2018). What is mood? A computational perspective. *Psychol. Med.* 48, 2277–2284. 10.1017/S0033291718000430 29478431 PMC6340107

[B12] Connolly J. P. (2016). *Principles of Organization of Psychic Energy Within Psychoanalysis: a Systems Theory Perspective.* thesis, Pretoria: University of South Africa. Unpublished doctoral thesis.

[B13] Connolly P. (2018). Expected free energy formalizes conflict underlying defense in freudian psychoanalysis. *Front. Psychol.* 9:1264. 10.3389/fpsyg.2018.01264 30072943 PMC6060308

[B14] Feldman R. (2012). Parent-infant synchrony: a biobehavioral model of mutual influences in the formation of affiliative bonds. *Monogr. Soc. Res. Child Dev.* 77 42–51. 10.1111/j.1540-5834.2011.00660.x

[B15] Fonagy P. Allison E. (2014). The role of mentalizing and epistemic trust in the therapeutic relationship. *Psychotherapy* 51 372–380. 10.1037/a0036505 24773092

[B16] Friston K. J. (2010). A free energy principle for the brain. *Nat. Rev. Neurosci.* 11 127–138. 10.1038/nrn2787 20068583

[B17] Friston K. Frith C. (2015a). A duet for one. *Conscious Cogn.* 36 390–405. 10.1016/j.cogn.2014.12.003 25563935 PMC4553904

[B18] Friston K. J. Frith C. D. (2015b). Active inference, communication and hermeneutics. *Cortex* 68 129–143. 10.1016/j.cortex.2015.03.025 25957007 PMC4502445

[B19] Friston K. Breakspear M. Deco G. (2012). Perception and self-organized instability. *Front. Computational Neurosci.* 6:44. 10.3389/fncom.2012.00044 22783185 PMC3390798

[B20] Friston K. Kilner J. Harrison L. (2006). A free energy principle of the brain. *J. Physiol.* 100 70–87. 10.1016/j.jphysparis.2006.10.001 17097864

[B21] Gendlin E. T. (1996). *Focusing-oriented Psychotherapy: a Manual of the Experiential Method.* New York, NY: Guilford Press.

[B22] Greenson R. R. (1967). *The Technique and Practice of Psychoanalysis.* Milton Park: Routledge.

[B23] Haken H. (1983/2004). *Synergetics: Introduction and Advanced Topics.* Berlin: Springer-Verlag. Original work published in 1983.

[B24] Haken H. (1993). *Advanced Synergetics: Instability Hierarchies of Self-Organizing Systems and Devices.* New York, NY: Springer-Verlag.

[B25] Hohwy J. (2016). The self-evidencing brain. *Noûs* 50 259–285. 10.1111/nous.12062

[B26] Holmes J. Nolte T. (2019). Surprise and the bayesian brain: implications for psychotherapy theory and practice. *Front. Psychol. Psychoanalysis Neuropsychoanalysis* 10:592. 10.3389/fpsyg.2019.00592 30984063 PMC6447687

[B27] Hopkins J. (2016). Free energy and virtual reality in neuroscience and neuropsychoanalysis: a complexity theory of dreaming and mental disorder. *Front. Psychol. Cogn. Sci.* 7:922. 10.3389/fpsyg.2016.00922 27471478 PMC4946392

[B28] Janoff-Bulman R. (1989). Assumptive worlds and the stress of traumatic events: applications of the schema construct. *Soc. Cogn.* 7:117. 10.1521/soco.1989.7.2.113

[B29] Keeney B. (1983). *Aesthetics of Change.* New York, NY: Guilford.

[B30] Kernberg O. F. (2004). “Borderline personality disorder and borderline personality organization: psychopathology and psychotherapy,” in *Handbook of Personality Disorders: Theory and Practice*, ed. Magnavita J. J. (Hoboken, NJ: John Wiley & Sons Inc), 92–119.

[B31] Kiverstein J. Rietveld E. Slagter H. A. Denys D. (2019). Obsessive compulsive disorder: a pathology of self-confidence? *Trends Cogn. Sci.* 23, 369–372. 10.1016/j.tics.2019.02.005 30954404

[B32] Koole S. L. Tschacher W. (2016). Synchrony in psychotherapy: a review and an integrative framework for the therapeutic alliance. *Front. Psychol.* 7:862. 10.3389/fpsyg.2016.00862 27378968 PMC4907088

[B33] Kruglanski A. W. (1989). *Lay Epistemics and Human Knowledge: Cognitive and Motivational Bases.* New York, NY: Plenum Press. 10.1007/978-1-4899-0924-4

[B34] Kuypers K. P. Ng L. Erritzoe D. Knudsen G. M. Nichols C. D. Nichols D. E. (2019). Microdosing psychedelics: more questions than answers? an overview and suggestions for future research. *J. Psychopharmacol.* 33 1039–1057. 10.1177/0269881119857204 31303095 PMC6732823

[B35] Lakatos I. (1970). “Falsification and the methodology of scientific research programmes,” in *Criticism and the Growth of Knowledge*, eds Lakatos I. Musgrave A. (Cambridge: Cambridge University Press), 91–196. 10.1017/cbo9781139171434.009

[B36] Leszczynski M. Chaieb L. Reber T. P. Derner M. Axmacher N. Fell J. (2017). Mind wandering simultaneously prolongs reactions and promotes creative incubation. *Sci. Rep.* 7:10197. 10.1038/s41598-017-10616-3 28860620 PMC5578971

[B37] Llewelyn S. Macdonald J. Aafjes-van Doorn K. (2016). “Process-Outcome studies,” in *APA Handbook of Clinical Psychology: Theory and Research*, eds Norcross J. C. VandenBos G. R. Freedheim D. K. (Washington, DC: American Psychological Association), 10.1037/14773-020

[B38] Mathys C. Daunizeau J. Friston K. J. Stephan K. E. (2011). A Bayesian foundation for individual learning under uncertainty. *Front. Hum. Neurosci.* 5:39. 10.3389/fnhum.2011.00039 21629826 PMC3096853

[B39] McNally R. Bryant R. Ehlers A. (2003). Does early psychological intervention promote recovery from posttraumatic stress? *Psychol. Sci. Public Interest* 4 45–79. 10.1111/1529-1006.01421 26151755

[B40] Mehrabian A. R. Lucas C. (2006). A novel numerical optimization algorithm inspired from weed colonization. *Ecol. Inform.* 1 355–366. 10.1016/j.ecoinf.2006.07.003

[B41] Mergenthaler E. (2008). Resonating minds: a school-independent theoretical conception and its empirical application to psychotherapeutic processes. *Psychotherapy Res.* 18 109–126. 10.1080/10503300701883741 18815969

[B42] Metzinger T. (2017). “Why is mind wandering interesting for philosophers?,” in *The Oxford Handbook of Spontaneous Thought: Mind-wandering, Creativity, Dreaming, and Clinical Conditions*, eds Fox K. C. R. Christoff K. (New York, NY: Oxford University Press), 97–112. 10.3389/fpsyg.2013.00746

[B43] Nerison R. M. Claiborn C. D. (1990). “Counselor attractiveness, similarity, and session impact: a field study,” in *Paper Presentated at the Annual Convention of the American Psychological Association* (Boston, MA).

[B44] Northoff G. (2018). *The Spontaneous Brain: from the Mind-Body to the World-Brain Problem.* Cambridge, MA: The MIT Press.

[B45] Palacios E. R. Razi A. Parr T. Kirchhoff M. Friston K. J. (2020). On Markov blankets and hierarchical self-organisation. *J. Theoretical Biol.* 486:110089. 10.1016/j.jtbi.2019.110089 31756340 PMC7284313

[B46] Park J. Mori H. Okuyama Y. Asada M. (2017). Chaotic itinerancy within the coupled dynamics between a physical body and neural oscillator networks. *PLoS One* 12:e0182518. 10.1371/journal.pone.0182518 28796797 PMC5552128

[B47] Parr T. Benrimoh D. A. Vincent P. Friston K. J. (2018). Precision and false perceptual inference. *Front. Integr. Neurosci.* 12:39. 10.3389/fnint.2018.00039 30294264 PMC6158318

[B48] Perls F. (1969). *Gestalt Therapy Verbatim.* Gouldsboro, ME: Gestalt Journal Press.

[B49] Peterfreund E. Schwartz J. T. (1971). Information, systems, and psychoanalysis: an evolutionary biological approach to psychoanalytic theory. *Psychol. Issues Monograph* 7 1–397. 4928130

[B50] Pezzulo G. Rigoli F. Friston K. J. (2018). Hierarchical active inference: a theory of motivated control. *Trends Cogn. Sci.* 22 294–306. 10.1016/j.tics.2018.01.009 29475638 PMC5870049

[B51] Polito V. Stevenson R. J. (2019). A systematic study of microdosing psychedelics. *PLoS One* 14:e0211023. 10.1371/journal.pone.0211023 30726251 PMC6364961

[B52] Rauchhaus R. (2009). Evaluating the nuclear peace hypothesis: a quantitative approach. *J. Conflict Resolution* 53 258–277. 10.1177/0022002708330387

[B53] Rogers C. (1959). “A theory of therapy, personality and interpersonal relationships as developed in the client-centered framework,” in *Psychology: A Study of a Science: Formulations of the Person and the Social Context*, ed. Koch S. (New York, NY: McGraw Hill).

[B54] Sadaghiani S. Hesselmann G. Friston K. J. Kleinschmidt A. (2010). The relation of ongoing brain activity, evoked neural responses, and cognition. *Front. Systems Neurosci.* 4:20. 10.3389/fnsys.2010.00020 20631840 PMC2903187

[B55] Schiepek G. Heinzel S. Karch S. Ploderl M. Strunk G. (2016). “Synergetics in psychology: patterns and pattern transitions in human change processes,” in *Self-organization in Complex Systems: The Past, Present, and Future of Synergetics, Understanding Complex Systems*, eds Pelster A. Wunner G. (Cham: Springer), 10.1007/978-3-319-27635-9_12

[B56] Schoeller F. Miller M. Salomon R. Friston K. J. (2021). Trust as extended control: human-machine interactions as active inference. *Front. Systems Neurosci.* 15:93. 10.3389/fnsys.2021.669810 34720895 PMC8548360

[B57] Seth A. Friston K. J. (2016). Active interoceptive inference and the emotional brain. *Philos. Trans. R. Soc. B* 371:20160007. 10.1098/rstb.2016.0007 28080966 PMC5062097

[B58] Smallwood J. Schooler J. W. (2015). The science of mind wandering: empirically navigating the stream of consciousness. *Annu. Rev. Psychol.* 66 487–518. 10.1146/annurev-psych-010814-015331 25293689

[B59] Stolorow R. Atwood G. Brandchaft B. (eds) (1994). *The Intersubjective Perspective.* Northvale, NJ: Jason Aronson.

[B60] Strupp H. H. (1993). The vanderbilt psychotherapy studies: synopsis. *J. Consult. Clin. Psychol.* 61 431–433. 10.1037/0022-006X.61.3.431 8326043

[B61] Tschacher W. Giersch A. Friston K. (2017). Embodiment and schizophrenia: a review of implications and applications. *Schizophr. Bull.* 43, 745–753. 10.1093/schbul/sbw220 28338892 PMC5472128

[B62] Tschacher W. Haken H. (2019). *The Process of Psychotherapy: Causation and Chance.* Cham: Springer.

[B63] Tyukin I. Tyukina T. Leeuwen C. (2009). Invariant template matching in systems with spatiotemporal coding: a matter of instability. *Neural Networks* 22 425–449. 10.1016/j.neunet.2009.01.014 19264447

[B64] Van de Cruys S. Evers K. Van der Hallen R. Van Eylen L. Boets B. de-Wit L. (2014). Precise minds in uncertain worlds: predictive coding in autism. *Psychol. Rev.* 121, 649–675. 10.1037/a0037665 25347312

[B65] Walter S. Schiepek G. K. Schneider S. Strunk G. Kaimer P. Mergenthaler E. (2010). The synchronization of plan activations and emotion-abstraction patterns in the psychotherapeutic process: a single-case study. *Psychotherapy Res.* 20 214–223. 10.1080/10503300903277437 19844842

[B66] Ward E. C. (2005). Keeping it real: a grounded theory study of African American clients engaging in counseling at a community mental health agency. *J. Couns. Psychol.* 52, 471–481. 10.1037/0022-0167.52.4.471

[B67] Zedelius C. M. Schooler J. W. (2015). Mind wandering “Ahas” versus mindful reasoning: alternative routes to creative solutions. *Front. Psychol.* 6:834. 10.3389/fpsyg.2015.00834 26136715 PMC4469818

